# Association between Environmental Dioxin-Related Toxicants Exposure and Adverse Pregnancy Outcome: Systematic Review and Meta-Analysis

**DOI:** 10.22074/ijfs.2015.4174

**Published:** 2015-02-07

**Authors:** Xinjuan Pan, Xiaozhuan Liu, Xing Li, Nannan Niu, Xinjuan Yin, Ning Li, Zengli Yu

**Affiliations:** 1 College of Public Health, Zhengzhou University, Zhengzhou, China; 2 Medical College, Henan University of Science and Technology, Luoyang, China

**Keywords:** Dioxin, Pregnancy Outcome, Meta-Analysis

## Abstract

Dioxin-related compounds are associated with teratogenic and mutagenic risks in laboratory animals, and result in adverse pregnancy outcomes. However, there were inconsistent results in epidemiology studies. In view of this difference, we conducted a systematic review and meta-analysis to examine this association and to assess the heterogeneity among studies. Comprehensive literature searches were performed to search for relevant articles published in English up to 15 May 2012. In total, we identified 15 studies which included 9 cohort and 6 case control studies. The Cochrane Q test and index of heterogeneity (I^2^) were used to evaluate heterogeneity. In either cohort studies (I^2^=0.89, p<0.0001) or case control studies (I^2^=0.69, p=0.02), significant heterogeneity of risk estimates were observed. Subgroup analyses found no significant increased risk of adverse pregnancy outcome with air dioxin-related compounds exposure (RR=0.99, 95% CI:0.85–1.16), no significant increased risk of spontaneous abortion (SAB) with exposure to food dioxin-related compounds (RR=1.05, 95% CI:0.80–1.37), higher significant risks of low birth weight (LBW) with exposure to food dioxin-related compounds (RR=1.55, 95% CI:1.24–1.94), and higher significant risks of birth defects with maternal solid contaminants dioxin exposure (OR=1.24, 95% CI:1.19–1.29). In conclusion, more evidences are needed to confirm the association between environmental dioxin-related compounds exposure and pregnancy outcome.

## Introduction

Many dioxin-related toxicants, including the polychlorinated biphenyls (PCBs), polychlorinated dibenzo-p-dioxins (PCDDs), and polychlorinated dibenzofurans (PCDFs) are persistent environmental contaminants. These compounds are characterized by high-affinity binding to the Ah receptor, which are thought to mediate most biological effects by the ligand-Ah receptor complex (1). Dioxin- related toxicants are ubiquitous contaminants of various industrial and combustion processes. They are extremely stable in the environment and have been classified as a type of known human carcinogen (2). In addition to cancer, dioxin-related toxicants may result in other health hazards, such as impaired or altered thyroid hormone regulation (3), immunological functioning (4), and neurological development (5). Furthermore, experimental studies indicate that exposure to tetrachlorodibenzo- p-dioxin (TCDD) is associated with increased teratogenic and mutagenic risks (6), and it has been linked to a variety of adverse pregnancy effects in animals, including spontaneous abortion (SAB) (7) and preterm birth (8).

Although reproductive effects of dioxin exposure have been reported in numerous experiments of animals, studies of this association in humans are limited, and the conclusions are always equivocal and often controversial. Some epidemiologic studies have demonstrated that exposure to dioxin-related compounds is associated with higher proportions of adverse outcomes including SAB, stillbirth and preterm delivery, fetal growth restriction, and low birth weight (LBW) (9-12). However other epidemiologic studies in humans have not shown such effects. Women in the United States who lived near a horse arena that had been sprayed with dioxin-contaminated oil did not have higher rates of fetal or infant mortality, intrauterine growth retardation, LBW, or birth defects compared with unexposed women (13). Studies conducted in a population living in contaminated areas, such as Vietnam veterans, exposed workers, and those affected by chloracne failed to show an association between TCDD exposure and birth defects (14-19).

The reasons for ambiguous findings in human studies are unknown but likely include the facts that many studies are limited by different methodologies, different endpoints, and small sample size (20). The present study is a meta-analysis relying on published studies in English to explore the relationship between exposure to dioxin-related compounds and adverse pregnancy outcomes. Additionally, our study has focused on general environmental dioxin exposure such as wood preservatives, consuming Swedish east coast fish, food contamination, municipal solid waste incinerator, and chemicals contaminated with TCDD, but not war-related Agent Orange contamination.

## Materials and Methods

We followed the Meta-analysis of Observational Studies in Epidemiology (MOOSE) criteria (21) for reporting. The data were extracted from published manuscripts, thus no research ethics board approval was necessary.

### Data source

Comprehensive literature searches were performed using the PubMed, Springer, Elsevier Digital Dissertations Databases, Scopus, and ISI web of knowledge for relevant articles published in English up to May 2012. Key words used were: "dioxin" or "TCDD" in conjunction with one of the following terms "pregnancy outcome", "reproductive outcomes", "pregnancy loss", "preterm delivery" , "spontaneous abortion", "SAB", "small for gestational age (SGA)", "SGA", "stillbirth", "low birth weight" and "LBW". We extended our search to review the reference list of retrieved articles and performed a manual search as a supplement. Two investigators carefully examined the full texts of the candidate articles to determine whether they met the inclusion criteria for the systematic review and meta-analysis.

The criteria for admitting articles to this study included: i. case-control studies or cohort studies; ii. data culled from studies in humans; iii. maternal or paternal exposure to dioxin-related compounds; and iv. preterm delivery (birth with gestation of less than 37 weeks); SAB (spontaneous loss of an intrauterine pregnancy at less than 20 weeks gestation); and stillbirth (fetal death that occurred at 20 weeks or greater gestation). LBW was defined as birth weight lower than 2500 g.

Case reports, letters, review articles, and abstracts without full texts were excluded from the analysis. Studies of military Agent Orange exposure that occurred during the Vietnam war and adverse pregnancy outcome in military were not considered because exposure of this population were higher than our focus here on environmental levels of dioxin.

When a study had duplicate publications, only the most inclusive publication was considered. For studies with multiple outcomes, only data concerning adverse pregnancy outcome were included in the analysis.

### Data extraction

Two investigators carefully and independently extracted the data. In case of inconsistent valuations, agreements were reached following discussion. For each study, the following characteristics were collected: first author, publication year, country, study period, characteristics of study population (exposed and unexposed/cases and controls), exposure definition/data source and measurement, exposed level, case ascertainment, and study results. The relative risks (RRs) or odds ratios (ORs) were extracted and summarized into a 2×2 table format.

### Bias assessment among included studies

Risks of biases in the eligible studies were assessed by two authors according to a checklist described by Shah and Balkhair (22). We evaluated the biases according to criteria for sample selection, exposure assessment, outcome assessment, confounder, analytical, and attrition. Different bias risk levels were classified in each category, which included unable to discern, no bias, low bias, moderate bias, and high bias.

### Statistical methods

Review Manager 5.0 (http://www.cc-ims.net/ RevMan.) was used for data synthesis and draw forest plots. Heterogeneity assumption between studies was examined by the Q-test and index of inconsistency (I^2^) (23). A random-effect model using the DerSimonian and Laird method was selected to pool data if there was signiﬁcant heterogeneity (p<0.05). Otherwise, the ﬁxed-effect model using the Mantel-Haenszel method was conducted. Publication bias was evaluated through the Begg’s test, the Egger asymmetry test, and visual inspection of funnel plots by Stata 8.0 (Stata Corporation, College Station, TX, USA). All p values were for the two-sided test and we considered p<0.05 as statistically signiﬁcant.

## Results

### Description of studies

**Fig 1:**
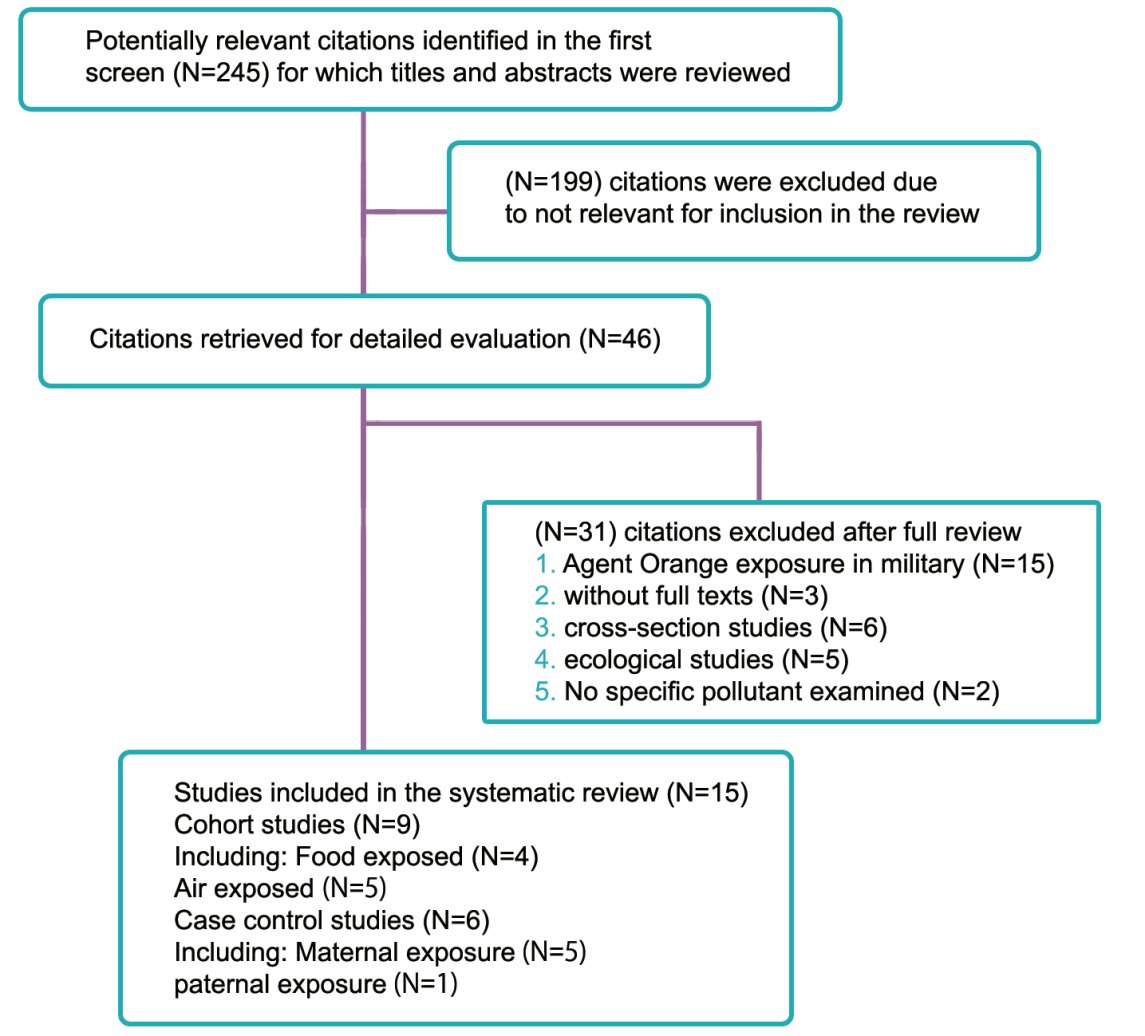
A flow diagram for selecting studies and specific exclusion reasons in this meta-analysis.

This review included 15 studies. Among the 15 studies identified, there were 9 cohort and 6 case– control studies. The results of the searches and the articles selection log are reported in figure 1. The characteristics of the 15 studies which included first author, year of publication, country, study period, characteristics of the study population, exposure definition/data source and measurement, exposed level, case ascertainment, and study results are reported in tables 1 and 2.

**Table 1:** Summary of cohort studies on the association between dioxin-related toxicants except Agent Orange and adverse pregnancy outcome


First author,year (Country)Study period	Exposed	Unexposed	Exposed level	Exposure defini-tion/data sourceand measurement	Caseascertainment	Results

**Rylander L,1995 (Sweden)1973-1991(24)**	65/1501Swedisheast coasts	106/3553 Swed-ish west coasts	No specificvalues	Food exposure:eat locallycaught fish/national Swed-ish populationregister	Low birthweights (<2500g), exclusion ofmultiple birthsand infants withmajor malforma-tions by SwedishMedical BirthRegister	High con-sumption ofcontaminatedfish from theBaltic Seaassociatedincreased riskfor low birthweight
**Rylander L,2000 (Sweden)1973-1993(25)**	Swedish eastcoast high intakeof POC contami-nated fish fromthe Baltic Sea	Swedish westcoast	No specificvalues	Food exposure:sisters to thesefishermen.Sisters whowere, or hadbeen, marriedto a fishermanwere excluded/national Swed-ish populationregister	LBW, SGA,Stillbirths, Earlyneonatal deaths(<7 days age),malformations,by SwedishMedical BirthRegister	Exposure toPOC duringchildhood andadolescenceincreasedthe risk ofLBW, but notaffect SGA,Stillbirths andother malfor-mations
**Small CM,2007 (US)1976-1997(26)**	529 women with1344 poten-tially exposedpregnancies in Michigan afterthe accidentalcontaminationof live stockscontaining PBBsand PCBs	All 861 womenreporting one ormore live birthsor spontaneousabortions inMichigan afterthe accidentalcontaminationof live stockscontaining PBBsand PCBs	PBB (ppb)Reference <1ppb; Exposed>1 ppb	Food exposure:food contamina-tion/exposures/based on therecords of Michi-gan Departmentof Public Health	Spontaneousabortions by self-reports	Results donot supportan associa-tion betweenexposure toPBBs or PCBsand risk ofspontaneousabortion
**Tsukimori K,2008 (Japan)1968-2004(11)**	122 pregnanciesbetween 1968-1977, 88 preg-nancies between 1978-1987, 98pregnanciesbetween 1988-2003	204 pregnan-cies before 1968when Yusho oilincident hap-pened	No specificvalues	Food exposure:the exposurereferring tothe Yusho oilincident/expo-sures based onthe records of theYusho studyinggroup	Spontaneousabortion, pretermbirth, preg-nancy loss andinduced abortionby self-reports	Only in preg-nancy in thefirst 10 yearsafter exposure,the propor-tions of in-duced abortionand pretermdelivery weresignificantlyincreasedcompared withthe propor-tions in preg-nancy before1968
**Vinceti M 2008 (Italy) 2003-2006(27)**	Person-years of 3796.64 women residing and 695.58 workersnear the municipal solid wasteincinerator	The remaining municipal population	0-10×10^-9^ μg/m^3^	Atmosphere exposure: according to mean annual atmospheric concentrations of, polychlorinated dibenzo-p-dioxin and dibenzofurans	Spontaneous abortion and birth defects by medical records	The study results provide little evidence of an excess risk of adverse pregnancy outcomes in women exposed to emissions from a modern municipal solid waste incinerator
**Karmaus W, 1995 (Germany) 1987-1988(28)**	49 exposed pregnancies	507 pregnancies unexposed	Median concentration was 0.5 pg/m^3^	Indoor air exposure: women working in daycare centers treated with wood preservatives in the State of Hamburg and its vicinity/employer’s liability scheme	Induced abortion, miscarriage, stillbirth, birth length and birth weight from mother’s health card	The significant differences between exposed and unexposed were 175 g in birth weight and 2 cm in length
**Fitzgerald EF,1989 (US) 1981-1984(29)**	482 persons who experience electrical transformer fire in Binghamton	The general population	TCDD average 3 ppm; TCDF: average 199 ppm	Air exposure: liability scheme exposure to the toxic contaminants of an electrical transformer fire/group exposure based on vital record	Spontaneous, fetal death, birth weight, congenital malformation from physician survey and hospital records	Infants with low birth weight or congenital malformations were similar to comparison population
**Mastroiacovo P,1988 (Italy) 1977-1982(30)**	2900 infants born between 1978 and 1982 near the accident	12391 infants born the same period not near the accident	A 192.8 μg/m^2^ B 3 μg/m^2^ R 0.9 μg/m^2^	Air exposure: live in zones A, B, R surrounding the factory and direct exposure to the accident/health surveillance program	Malformation and birth defects by medical records	Failed to demonstrate any increased risk of birth defects associated with TCDD
Schnorr TM,*2001 (US)1950s-1960s (31)	247 wives of 281 workers who were exposed to chemicals contaminated with TCDD; 632 pregnancies to workers’ wives	215 wives of the referents; 707 pregnancies to referents’ wives	Serum TCDD level, exposed254 ppt;referent: 6 ppt;	Paternal exposure: Occupational exposures (chemical workers who were exposed to TCDD)/ exposures based on NIOSH’s records	Data on spontaneous abortion and sex ratio by (recognized clinical pregnancies) self-reports	Not find an association between paternal serum TCDD level and spontaneous abortion or sex ratio of offspring in this population


*; Study was not used in meta-analysis because the objects in this study were fathers, POC; Persistent organochlorine compounds, PCB; Polychlorinated biphenyls, PBBs; Polybrominated biphenyls, NIOSH; National Institute for Occupational Safety and Health, TCDD; etrachlorodibenzo-p-dioxin, TCDF; Tetrachlorodibenzofuran, LBW; Low birth weight and SGA; Small for gestational age.

**Table 2:** Summary of case control studies on the association between dioxin-related toxicants except Agent Orange and adverse pregnancy outcome


First author year(country) studyperiod	Exposed	Unexposed	Exposed level	Exposed defini-tion/data sourceand measurement	Caseascertainment	Results

**Dimich-ward H,1996 (Canada)1952-1988(32)**	4302 cases oflow birth weight,prematurity,stillbirths, orneonatal deaths.And 942 otherbirth defect cases	5 referentsmatched percase accordingto year of birthand gender	Cumulative hours ofexposure tochlorophenates	Paternal exposure: occupational exposures(worked forsawmills wherechlorophenatewood preserva-tives had beenused)/, based onpersonal records	All types ofbirth defects byself-reports andexaminations	No associations werefound for lowbirth weight,prematurity,stillbirths, orneonataldeaths. expo-sure increasedthe risk fordevelopingcongenitalanomalies ofthe eye andanencephalyor spina bifidaandcongenitalanomalies ofgenital organs
**Orr M,2002 (US)1983-1988(33)**	13938 minority infants withmajor structuralbirth defectswhose mothersresided in se-lected countiesat the time ofdelivery	14463 minorityinfants withoutbirth defectwho were ran-domly selectedfrom the samebirth cohortas the casesubjects	No specificvalues	Maternal exposure: environmental pollution(shared thesame tract as thehazardous wastesites during thetime of delivery)/exposures basedon the data listedin EPA’s comput-erized database	All types ofbirth defectsby CBDMP’srecords	Potentialexpose tolow vola-tile organiccompounds as-sociated withanencephaly
Eskenazi B,*2003 (US)1996-1998(17)	Spontaneousabortions, andsmall for gestational age in 888total pregnancies	Not spontaneous abortions,and not smallfor gestationalage in 888 totalpregnancies	MaternalSerum TCDDlevels: median(IQR) 46.6 ppt(24.3–104.0)	Maternal exposure: Chemicalfactory explosion/exposuresbased on formerstudy’s records	Spontaneousabortions, birthweight, andsmall for gestational age byself-reports andmedical reports	There was no association of log10 TCDDwith SAB,with birthweight, orwith SGA
**Kuehn CM,2007 (US)1997-2001(34)**	63006 infantswith malformations occurrencesin WashingtonState	315030 infantsrandomlySelectedwithout malformations inWashingtonState during thesame years	No specificvalues	Maternal exposure: Distancebetween maternal residenceand nearesthazardouswaste site wasmeasured usingGIS software. /exposues basedon CSCS Reportconducted by theWADOE	All types ofmalformationsby hospital discharged reportsoffered by theBERD	Relative to living >5 milesfrom a site,living <5 mileswas associatedwith increasedrisk of anymalformationsin offspring
**Vinceti M,2009 (Italy)1998-2006(35)**	228 births and induced abortions with diagnosis of congenital anomalies in a community resides in the city of Reggio Emilia (Italy), in which a municipal solid waste incinerator with a capacity of . 70,000 tons/y is located	A randomly selected living birth without diagnosis of malformations during the same year to women residing in the Reggio Emilia municipality , referred to the same hospital and born in the same year of the matched "case" mother	0.5~1.0 ug/m³	Maternal exposure: exposure to the emissions from a municipal solid waste incinerator/exposures based on estimation with the support of GIS data	All types of malformations by records form RMER and the Eurocat program	Do not lend support to the hypothesis that the environmental contamination occurring around an incineration plant may induce major teratogenic effects
Cordier S,*2010 (France)2001-2003(36)	304 infants with urinary tract birth defects diagnosed in the Rhône-Alpes region	A random sample of 226 population controls frequency-matched for infant sex and year and district of birth.	Median exposures were 3.0×10^-3^ pg/m³ and1.7×10^-5^ pg/m³, respectively	Maternal exposure: Exposure to dioxin in early pregnancy at the place of residence,/exposures based on records of the operator or a public body, during the relevant time period, yet on a global metal emission score assignned by an expert group	All types of urinary tract birth defects by self-reports	Risk was increased for mothers exposed to dioxin above the median (OR 2.95, 95% CI:1.47 to 5.92)


*; Study was not used in meta-analysis because of no available data (Dimich-ward et al. (32), Eskenazi et al. (17)) or limitation in special birth defects (Cordier et al. (36)). CBDMP; California birth defects monitoring program, GIS; Geographic information systems, CSCS; Confirmed and suspected contaminated sites report, WADOE; Washington state department of ecology, BERD; Birth events records database, RMER; Registry of congenital malformations of the Emilia-Romagna Region, EPA; Environmental Protection Agency, TCDD; Tetrachlorodibenzo-p-dioxin, SAB; Spontaneous abortion and SGA; Small for gestational age.

### Quality of included studies

The results of bias assessment of the included studies are shown in table 3. From the 15 studies, 6 had an overall moderate risk of bias and 9 had a low risk of bias. Moderate risk of bias was assigned mostly due to indirect exposure assessment methods used in these studies (22). Various exposure sources and exposure styles were reported on by the different studies, however, the majority exposure components were dioxin or dioxin-like compounds.

### Test of heterogeneity

Review Manager 5.0 was used to test the heterogeneity of the 9 cohort and 6 case-control studies. In either the cohort (p<0.0001) or case– control (p=0.02) studies, we noted significant heterogeneity of the risk estimates as shown in table 4. The I^2^ was 0.89 for cohort studies and 0.69 for case-control studies.

**Table 3:** Risk of bias assessments of included studies


Authors	Confoundersadjusted	Risk of biases						
		Selection	Exposureassessment	Outcomeassessment	Confounderadjustment	Analytical	Attrition	Overall

**Rylanderet al. (24)**	Year ofbirth,gender, ma-ternal age, parity,marital status,and smokinghabits in earlypregnancy	Low	Moderate	Low	None	None	Can’t tell	Moderate
**Rylanderet al. (25)**	Gender, maternalage, parity,andsmoking habitsin early preg-nancy	Low	Moderate	Low	None	None	None	Moderate
**Smallet al. (26)**	Maternal age atconception, ageat menarche, andprior infertility	Moderate	None	Low	Low	None	None	Low
**Tsukimoriet al. (11)**	Age at delivery	Low	None	Low	Low	None	Low	Low
**Vinceti et al. (35)**	Age and calendar year	Low	Moderate	None	Low	Low	Low	Moderate
**Karmauset al. (28)**	Height andweight of themothers, occupa-tional conditions, smoking, alco-hol consumption,gestational age,parity, complica-tions	Low	Low	None	None	None	None	Low
**Fitzgeraldet al. (29)**	Age ,occupation,sex, race	None	Low	None	Low	Low	None	Low
**Mastroiacovoet al. (30)**	Prenatal history,birth order, parental age, parental occupation,parental chronicdiseases, andfamily history	Low	Low	None	Low	Low	Can’t tell	Low
**Schnorret al. (31)**	Maternal age,Hispanic ethnic-ity, and thyroiddisease medication, mother’seducation andfather’s race.	Low	None	Low	None	None	Moderate	Low
**Dimich-wardet al. (32)**	Gender, mother’sage, father’s age,birth year	Moderate	Low	Low	Low	None	Can’t tell	Moderate
**Orret al. (33)**	Racial/ethnic group, child’s sex, maternal age, prenatal care	Low	Moderate	Low	Low	Low	Low	Moderate
**Eskenaziet al. (17)**	Maternal age,education, maternal smoking, and alcohol use, previous parity, history of low birth weight, and spontaneous abortion, body mass index, height, maternal weight gain, gestational age, infant’s sex, and years from pregnancy tointerview	Low	None	None	None	None	Low	Low
**Kuehnet al. (34)**	Maternal and paternal age, maternal smoking and alcohol consumption during pregnancy, prior fetal death, race/ethnicity, maternal education, marital status, parental employment, urban vs. rural residence.	None	Moderate	None	None	None	Low	Moderate
**Vincetiet al. (35)**	Maternal age and education	Low	Low	Low	Low	None	Low	Low
**Cordieret al. (36)**	Infant sex, year, district of birth, socioeconomic characteristics, mother’s residence, maternal age, parental geographical origin, educational level, employment status, treatment for chronic disease, folic acid supplementation, history of urinary tract birth defects, parity, obesity, tobacco and alcohol use, family history	Low	Low	Low	none	None	Low	Low


**Table 4:** Summary of estimates of risk and heterogeneity in overall and sub-group analysis


Subgroup	Numbers ofstudies	Summary ORor RR (95% CI)	Measure of heterogeneity			Analysis
			Q-value	P value	I^2^	Model

Study design						
Cohort studies	9	1.23 (0.91, 1.67)	69.63	0.0001	0.89	RE
Case control studies^A^	4	1.30 (1.09, 1.56)	9.56	0.02	0.69	RE
**Cohort studies**						
Air dioxin-related toxicantsexposure and adversepregnancy outcome	4	0.99 (0.85, 1.16)	4.17	0.24	0.28	FE
**Food dioxin-related toxicantsexposure and**						
Spontaneous abortion	2	1.05 (0.80, 1.37)	1.30	0.25	0.23	RE
Low birth weight	2	1.55 (1.24, 1.94)	0.41	0.52	0	RE
**Case control studies**						
Maternal solid contaminantsdioxin exposure and birth defects	3	1.24 (1.19, 1.29)	1.52	0.47	0	FE


RE; Random-effect model, FE; Fixed-effect model, I^2^; Index of inconsistency and A; Cannot extract effective data from two studies [Dimich-Ward et al. (32), Eskenazi et al. (17)].

### Subgroup analysis

**Fig 2:**
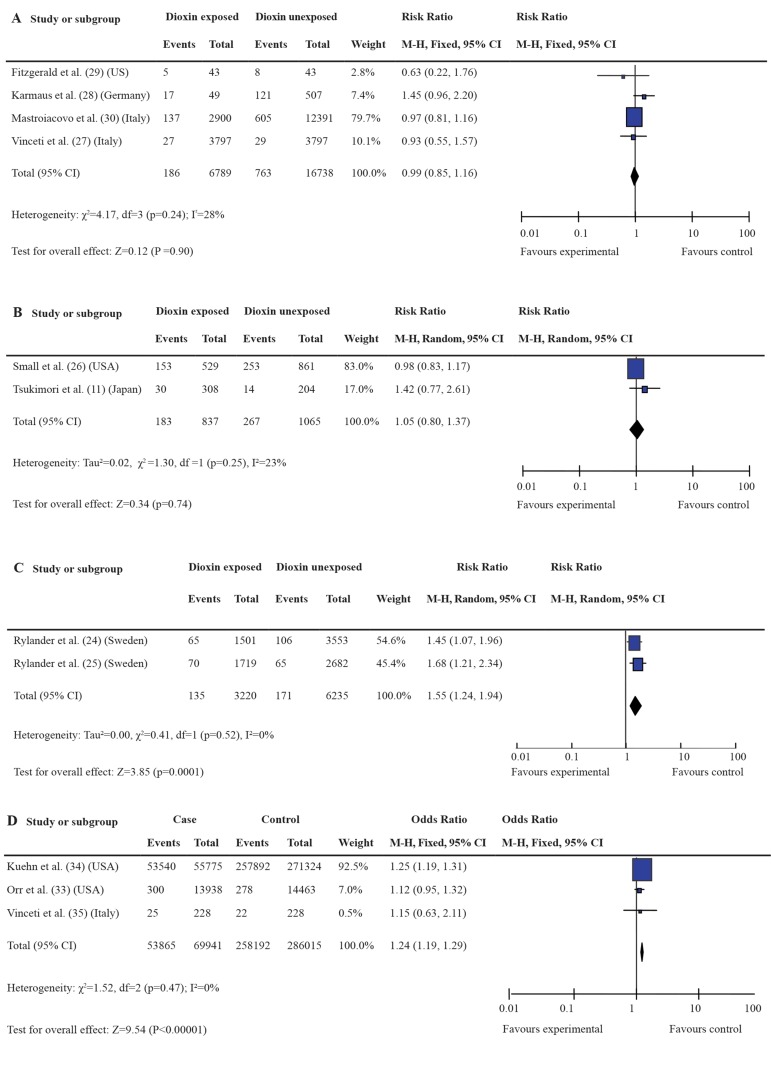
Forest plots for subgroup analysis. A. Forest plots for the association between air dioxin exposure and adverse pregnancy outcome in cohort studies. B. Forest plots for the association between food dioxin exposure and spontaneous abortion in cohort studies. C. Forest plots for the association between food dioxin exposure and low birth weight in cohort studies. D. Forest plots for the association between maternal solid contaminants dioxin exposure and birth defects in case control studies.

Significant heterogeneities were observed in both cohort and case control studies, which could possibly be attributed to the differences in population under investigation, exposure source, exposed level, and pregnancy outcome. Thus, further subgroup analyses were needed. The heterogeneity tests of subgroups are shown in table 4. As seen in table 4 and figure 2A-D, we found no significant increased risk of adverse pregnancy outcome with exposure to air dioxin-related compounds (RR=0.99, 95% CI:0.85-1.16, Fig 2A). There was no significant increased risk of SAB with food dioxin- related compounds (RR=1.05, 95% CI: 0.80- 1.37, Fig 2B). However there was a significantly increased risk noted in LBW to food dioxin-related compounds (RR=1.55; 95% CI: 1.24-1.94, Fig 2C) and in birth defects with maternal exposure to solid contaminants dioxin (OR=1.24; 95% CI:1.19- 1.29, Fig 2D).

### Sensitivity analyses and publication bias

Sensitivity analyses were conducted to assess whether each individual study affected the final results. These analyses suggested that no individual study affected the results in all subjects using the exclusion method step by step (data not shown).

**Fig 3:**
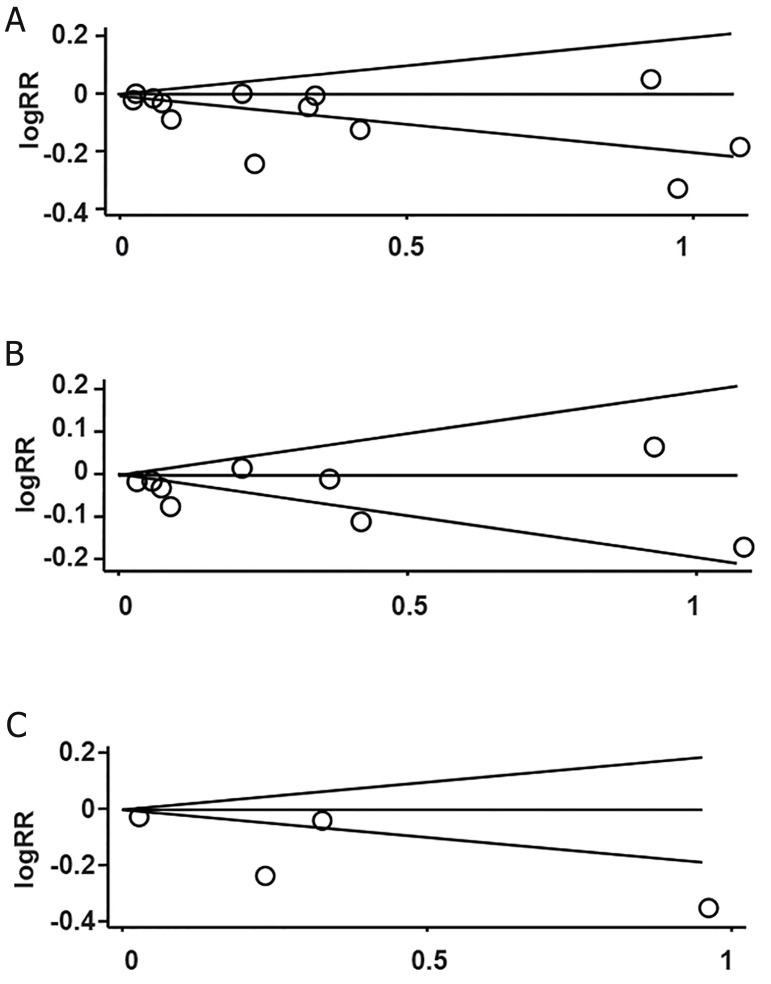
Funnel plots for dioxin exposure and adverse pregnancy outcomes (Begg’s funnel plot with pseudo 95% confidence limits). A. overall studies, B. cohort studies and C. case control studies.

Funnel plots of all studies revealed no asymmetrical distribution of ORs or RRs (Fig 3 A-C), which suggested no significant publication bias in the overall studies (Egger’s test: t=-1.98, p=0.073). When studies were stratified by study design, the funnel plots and Egger’s test also indicated no publication bias among either case-control (Egger’s test: t=-0.98, p=0.360) or cohort (Egger’s test: t=-1.72, p=0.228) studies.

## Discussion

### Principal findings

In this systematic review of 15 studies, we identified variable effects of exposure to dioxin-related compounds on adverse pregnancy outcome which included LBW, SAB, SGA, stillbirth, and birth defects. There was an association between exposures to food dioxin-related toxicants with LBW; maternal exposure to solid contaminants dioxin was associated with birth defects. The association between exposure to dioxin-related toxicants and other adverse pregnancy outcomes was inconclusive. Hence, investigation of the effect of dioxin exposure on adverse pregnancy outcomes is challenging, and further studies with improved methodologies are needed to establish or refute an associative relationship.

In 2002, Ngo et al. (37) systematically reviewed studies of parental exposure to Agent Orange, dioxin- contaminated defoliants which used in Vietnam War, which appeared to be associated with an increased risk of birth defects. However, their study had a significant heterogeneity of effects across study populations. Their conclusion was based on 11 Vietnamese cohort and cross-sectional studies and 6 non-Vietnamese cohort studies, which suggested that the subjects had a specific higher-level of dioxin exposure. In their 2008 systematic review of 7 studies about the association between paternal exposure to Agent Orange and spina bifida, Ngo et al. (38) concluded that paternal exposure to Agent Orange was associated with increased risk of spina bifida, however when analyzed according to the study design, the association was not statistically significant for the cohort studies.

### Strength and weakness of the review

Epidemiologic studies of the association between exposure to TCDD or related compounds (e.g., other dioxins, furans, and dioxin-like PCBs) and pregnancy outcome in humans are inconsistent - probably due to limitations incurred by inadequate methodology, inappropriate endpoints, and small sample size, among other reasons. However, meta-analyses have the advantages that increase statistical power by pooling the results from small individual studies and also permit examination of the variability between studies (39).

This review also has some limitations. We restricted our searches to English publications due to the scope of information that might not be available in other languages. We did not include gray literature, abstracts, conference articles, and proceedings in this systematic review. The methodology quality of included studies was assessed solely according to the description by Shah and Balkhair (22). However, a number of internal validity or "risk of bias" tools have been developed for observational studies (40-43) and the Agency for Healthcare Research and Quality has released recent guidance on this topic (44). In addition, none of the included studies assessed impact based on only TCDD toxicity. Research on the health effects of TCDD needs to consider not only TCDD but also other factors such as moisture, temperature and nutrition, etc. (22, 45, 46). Thus, it is important to take into account these limitations when considering the conclusions of this review.

### Potential non-causal explanations

There were likely multiple reasons for the failure to find an association between exposure to dioxinrelated compounds and adverse pregnancy outcomes in humans.

Compared to experimental studies, investigative studies in humans are not like adult animals who are better equipped to combat dioxin exposure. Mocarelli et al. (47) described a permanent reduction in sperm quality in men exposed to TCDD prior to puberty. Numerous studies have demonstrated that males can confer a risk of preterm birth, pre-eclampsia and other adverse pregnancy outcomes to their partners, (48, 49) though the mechanisms have not been established. However, animal models clearly demonstrate that early life (in utero) toxicant exposures do have adult reproductive effects (50, 51). Thus, there exists the possibility in humans that the timing of exposure is critical, but difficult to assess with regard to subsequent pregnancy outcome.

Second, but equally important, emerging studies indicate that TCDD exposure alters the impact of a subsequent environmental stressor (i.e., infection) (52). Therefore, a TCDD associated adverse outcome in pregnancy may not be noted if the mother is otherwise healthy, but may only become a risk if a secondary stressor is present. Additionally, adverse pregnancy outcome such as LBW, SAB or SGA have varied and multiple etiologies and pathogenesis.

Potential bias such as selection and measurement bias, confounding, and publication bias exist in all meta-analyses, particularly in observational studies.

Because of different exposure states, different exposure sources, commonality of exposure, and the lack of using biomarkers to measure individual exposure, it is possible that some individuals had either minimal or no TCDD exposure level, which might entail further exposure misclassification bias. In some studies, the assessment of exposure was made based on groups of study participants according to their residential location, workplace or intake of TCDD contaminated food history. Some studies used the address at the time delivery to characterize a mother’s exposure and did not take into account a mother’s residential history, which might also lead to exposure misclassification. Based solely on one single address, the exposure estimated in those studies could only partially reflect an individual’s true exposure. Furthermore, the assessment of exposure and outcome in some studies has been made largely through interviewing parent(s) at the time of data collection, which generally occurred 10 years or more after exposure. This approach is known to miss those who died in both exposed and non-exposed groups, and can introduce survival bias. Some other studies identified only certain specific, but not all, adverse pregnancy outcomes which were of interest. These methods of data collection inevitably excluded malformed cases, which were not known by their parents. This type of bias would have underestimated, not overestimated, the risk of dioxin and adverse pregnancy outcome.

### Possible mechanism

It is impossible to explain the mechanism of the association between exposure to dioxinrelated toxicants and adverse pregnancy outcomes in this systematic review. However, a large number of previous animal studies indicate that TCDD is associated with a developmental syndrome that involves hydronephrosis, cleft palate, and fetal thymic atrophy in mice (53), and increased fetal loss and reduction in birth weight in experimental studies in rodents and monkeys (7, 54-58). Meanwhile, large experimental studies demonstrate that the toxic effects of TCDD are mediated by aryl hydrocarbon receptor (AhR). TCDD is the most potent activator of AhR. The activated AhR has been described to cause toxic effects reminding symptoms of vitamin A depletion such as respiratory tract and bile duct keratinization, dermal and epithelial lesions, thymus atrophy, immunodeficiency or impaired reproduction (59, 60). Some studies suggest that there are interactions between AhR ligands and the retinoid transport system, metabolism and signaling because it has been described that at least some of the negative effects caused by AhR ligands in TCDD exposed animals can be compensated by supplementation with vitamin A (59, 61). We speculate that the reason of intaking TCDD contaminated fish and Yusho oil had no association with SAB maybe partly because fish and oil are rich in vitamin A.

## Conclusion

The association between exposure to environmental dioxin-related toxicants, with the exception of Agent Orange, and pregnancy outcome is inconclusive. Thus, examination of dioxin exposure and pregnancy outcome is challenging. The biological mechanism of this association and methodological limitations of the studies warrant the consideration of conducting large-scale, well-designed studies in the future. Future studies need to include biological measures of exposure.
